# RhoB supports rubella infection and impairs endothelial barrier integrity through downstream ROCK signaling

**DOI:** 10.1186/s12964-026-02898-w

**Published:** 2026-04-27

**Authors:** Igor Kovacevic, Marie Müller, Leonie Mair, Vivien Henschke, Annkatrin Kowalski, Jes-Niels Boeckel, Karolin Kropf, Guido Posern, Uta Reibetanz, Claudia Claus

**Affiliations:** 1 Institute of Physiological Chemistry, Medical Faculty, Martin Luther University Halle-Wittenberg, Halle, Germany; 2 Institute of Medical Microbiology and Virology, Medical Faculty, Leipzig University, Leipzig, Germany; 3 Klinik und Poliklinik für Kardiologie, Universitätsklinikum Leipzig, Leipzig, Germany; 4 Institute for Medical Physics and Biophysics, Medical Faculty, Leipzig University, Leipzig, Germany

## Abstract

**Background:**

During infection, alterations of the endothelium’s barrier function are multifaceted and virus-specific. Rubella virus (RuV) infection during pregnancy damages the endothelium in the placental and fetal vasculature, thereby contributing to the development of congenital rubella syndrome. However, the extent and mechanisms behind RuV-induced endothelial barrier disruption have not been previously documented.

**Methods:**

To investigate if RuV directly impairs endothelial barrier integrity, we infected primary human umbilical vein endothelial cells and analyzed the barrier integrity using Electrical Cell-Substrate Impedance Sensing (ECIS). Furthermore, we applied fluorescence microscopy to analyze RuV-induced changes in cell morphology, the actin cytoskeleton, and cell–cell junctions. To dissect the molecular mechanisms behind the observed alterations induced by RuV infection in endothelial cells, we determined levels of RhoA and RhoB GTPases, followed by depletion of both proteins using siRNA transfection and application of pharmacological inhibitors of the Rho/ROCK signaling axis.

**Results:**

Rubella virus infection in endothelial cells induced a comparatively low level of cytopathogenicity, accompanied by an elongated morphology. Most notably, a reduction in endothelial barrier integrity occurred at later time points of infection, as measured by ECIS. While the overall expression levels of junctional proteins appeared unaffected, membrane localization of zonula occludens-1 (ZO-1) was significantly reduced. This was accompanied by a marked increase in actin stress fibers and enhanced RhoB GTPase levels. The siRNA-mediated downregulation combined with time-dependent inhibitor application confirmed a contributory role for RhoB to RuV infection at early post-entry steps. The elongated morphology of infected endothelial cells was associated with the chemokine CCL5. Notably, cell elongation induced by treatment with supernatant from infected cells was insufficient to impair the barrier integrity in the absence of productive RuV replication. In comparison, depletion of RhoB expression or ROCK inhibitor application revealed the involvement of the RhoB/ROCK signaling axis in the impairment of barrier integrity and induction of stress fibers by RuV infection. Additionally, altered localization of ZO-1 was restored after application of ROCK inhibitor Y-27632 to uninfected control levels.

**Conclusions:**

Collectively, our data highlight the involvement of Rho GTPase–ROCK signaling in alterations of endothelial cell actin dynamics and barrier integrity in rubella virus pathology.

**Supplementary Information:**

The online version contains supplementary material available at 10.1186/s12964-026-02898-w.

## Introduction

The endothelium serves as a crucial barrier against viral infections, limiting virus dissemination through the bloodstream and restricting its spread within the host. Both cell-associated and cell-free passage across the endothelium precede viremia, enabling subsequent infection of target tissues. Among viruses with a viremic phase together with endothelial involvement, rubella virus (RuV) is a prominent example. In our previous work, we demonstrated that RuV infection diminishes the angiogenic capacity of endothelial cells via type I interferon (IFN)-induced production of the cytokine CXCL10 [1]. This in vitro evidence shows similarities to some of the clinical observations, such as villitis and endothelial necrobiosis in placental tissue from mothers with primary rubella during pregnancy, as well as reduced placental weight [2]. Fetal transmission, particularly during the first trimester of pregnancy, can lead to congenital rubella syndrome (CRS) in the infant. Among the causes of hearing loss, a common symptom of CRS, are a reduced stria vascularis and a decreased number of capillaries with signs of cellular damage [3, 4]. Thus, the endothelium plays a role in the pathogenesis of postnatal and perinatal rubella. Additionally, a prevailing hypothesis for RuV transmission from mother to fetus suggests that desquamated endothelial cells may act as virus carriers to the fetal system [4].

Congenital rubella pathology does also include neurological symptoms and RuV replication in the brain [5], Moreover, endothelial involvement in rubella-associated neuropathogenicity was observed in a rare case of fatal encephalitis in a child with a homozygous mutation in the IFN-α/β receptor (IFNAR2) following vaccination with the live attenuated measles, mumps, and rubella (MMR) vaccine. In this case, both mumps and RuV were detected in brain biopsy sections [6]. This indicates that the in the absence of protective and antiviral type I IFN response, attenuated vaccine strain can traverse the blood–brain barrier, of which endothelial cells are a key structural component. However, it is unknown whether RuV infection disturbs endothelial barrier integrity and if so, how. The integrity of the endothelial barrier is influenced by actin cytoskeleton dynamics contributing to the formation and stability of cell–cell junctions [7]. For RuV infection a prominent redistribution of actin filaments was reported in two animal cell lines, namely Vero and BHK21 [8, 9]. Rho GTPases and especially RhoA and RhoB are key regulators of cell adhesion and cytoskeleton dynamics and as such of endothelial barrier integrity [10–12]. Rho GTPases respond to external stimuli, including viral infections, and play roles in modulating endothelial permeability [7, 10]. We hypothesized that RuV infection alters the endothelial barrier in association with components of the cytoskeleton–cell–cell junctions complex. To address this, we used human umbilical vein endothelial cells (HUVEC) as an endothelial infection model for electric cell-substrate impedance sensing (ECIS) together with siRNA-mediated downregulation of RhoA and RhoB and time-dependent application of inhibitors targeting Rho GTPases and ROCK (Rho-associated protein kinase). We observed that in particular RhoB supported virus infection at an early post-entry step and together with downstream ROCK contributed to actin re-distribution and endothelial barrier impairment at later stages of infection. Inhibition of ROCK signaling was sufficient to restore ZO-1 localization at the cell surface to levels comparable to the uninfected control. In contrast, infection-associated cell elongation was related to factors secreted by infected cells, namely the CCL5 chemokine. Notably, this morphological change did not impair endothelial barrier integrity per se. In conclusion, we identified a contributory role of the RhoB-ROCK signaling axis in RuV-induced dysfunction of HUVECs. These endothelial changes are consistent with a central role of endothelial cells in rubella pathogenesis in vivo.

## Material & methods

### Cell lines and virus infection

Human umbilical vein endothelial cells (HUVEC) from pooled donors were purchased from Lonza, (#C2519A) and maintained in endothelial cell growth basal medium (EBMTM, #CC-3121, Lonza) with 10% fetal bovine serum (FBS), 0.1% hydrocortisone, 30 μg/mL gentamicin sulfate, 15 ng/mL amphotericin (GA1000), 70 μM ascorbic acid, 3 ng/mL human epidermal growth factor (hEGF) and 12 μg/mL bovine brain extract (maintenance medium) or endothelial cell medium (ScienCell, Cat#1001) with 5% FBS and humidified atmosphere with 5% CO_2_ at 37 °C for up to five passages. Cell passaging was initiated at confluency with 0.1% trypsin–EDTA. Human kidney epithelial cells (HK-2), (CRL-2190, ATCC) were grown in Dulbecco’s modified eagle medium (DMEM) supplemented with 10% FCS and 1 mg/ml D-Glucose. To generate RhoB knockout (KO) HK-2 cells, lentivirus carrying the sgRNA sequences targeting RhoB was produced. For this purpose RhoB-targeting sgRNAs were cloned into the lentiCRISPR-V2 vector (Addgene; Plasmid # 52,961, a gift from Feng Zhang), [13] and packed into lentiviral particles. Cas9 control cells only expressed the empty lentiCRISPR-V2 vector (more detailed characterization of RhoB KO HK-2 cells will be published elsewhere).

### Inhibitors and cytokines

Recombinant human interferon (IFN)-β (Cat#300-02BC, PeproTech, Thermo Fisher Scientific) was used at 10 ng/ml, the ROCK1/2 inhibitor Y-27632-dihydrochlorid (Y-27632, Sigma-Aldrich, Merck) at 10 µM and the Rho Inhibitor I as a highly purified C3 transferase (C3, #CT04, Cytoskeleton Inc.) at 1 µg/ml. The inhibitors were applied for indicated incubation times followed by medium change (wash-out). IFN-β was maintained during cell culture without wash-out.

### Antibodies

Mouse anti-rubella monoclonal antibody (mAb), to E1, clone EI-20 (#MAB925, Sigma-Aldrich, Merck) and to capsid (C) protein (clone 2–36, #C66496M and #10192, Meridian Life Science [Meridian Bioscience]) were used for immunofluorescence analysis at a dilution of 1:200, 1:100 and 1:200, respectively. MAB925 was used for western blot at a 1:2,000 dilution. VE-Cadherin (D87F2) XP® rabbit mAb (#2500, Cell Signaling Technology) was used at a dilution of 1:400 for immunofluorescence analysis and 1:1,000 for western blot. Rabbit anti-ZO-1 polyclonal antibody (#21773–1-AP, Proteintech, Thermo Fisher Scientific) was used at a dilution of 1:200 for immunofluorescence and 1:2,000 for western blot. Rabbit anti-pMLC2 (#3671, Cell Signaling) and mouse anti-ZO-1 Alexa 488 (ZO1-1A12, #33-9100, Thermo Fisher Scientific) were used in immunofluorescence analysis at a 1:100 dilution. The neutralizing polyclonal goat IgG antibody (Cat#AF-278-NA, Bio-Techne) targeting human CCL5/RANTES was used at 0.1 µg/ml and applied 8 and 20 h after RuV infection. As isotype control (Ctrl), normal polyclonal goat IgG antibodies (Cat#AB-108-C, R&D Systems, Bio-Techne) were used. For western blot analysis rabbit anti-cleaved caspase-3 (Asp175), (5A1E), (Cat#9664, Cell Signaling Technology), rabbit anti-ERK2 (K-23), (Cat.#sc-153, Santa Cruz Biotechnology), rabbit anti-GAPDH D16H11 XP (#5174S, Cell Signaling Technology), anti-RhoA (67B9) rabbit mAb, (#2117, Cell Signaling Technology) and anti-RhoB (D1J9V) rabbit mAb (#63876, Cell Signaling Technology) were used as primary antibodies at a dilution of 1:1,000. As secondary antibodies goat anti-rabbit IgG, HRP-linked antibody and horse anti-mouse IgG, HRP-linked antibody (#7074 and #7076, respectively, Cell Signaling Technology) were used at a dilution of 1:5,000.

### Infection with rubella virus

For infection experiments the RuV clinical isolate RVi/Wuerzburg.DEU/47.11 (Wb-12), genotype 2B (kindly provided by Dr. B. Weißbrich, University of Wuerzburg) was used at a multiplicity of infection (MOI) of 10. 3 h, 24 h or as indicated after plating of 7 × 10^4^ HUVEC per well of a 24 well plate, 200 µl of virus inoculum were added. After an incubation of 2 h, medium change after PBS wash step was carried out.

### UV inactivation of virus particles

For the application of UV-inactivated supernatants, cell culture supernatants were collected at 72 hpi and centrifuged at 200 × g for 5 min. UV inactivation was carried out for virus particle preparations (RuV_UV_) and supernatants (mock_UV_SN and RuV_UV_SN) at a dose of 900,000 μJ/cm^2^ in a UV Crosslinker (Stratalinker 2400). Hereafter, 200 µl of supernatants were added per well of a 24 well plate to 7 × 10^4^ HUVEC 24 h after plating. Cells were incubated for four hours, followed by a medium change. Subsequently, cells were further incubated for 2 days prior to analysis.

### Cell viability assay

Cell viability was assessed using the enhanced cell counting kit-8 (CCK-8, Elabscience) according to the manufacturer’s instructions. A formazan dye results from the reduction of the WST-8 salt in the CCK-8 solution, which depends on cellular dehydrogenases. The CCK-8 solution was added at 72 hpi followed by an incubation for 2 h, after which absorbance (optical density [OD]) was measured at 450 nm with a reference wavelength set at 650 nm using a Tecan microplate reader.

### Immunofluorescence analysis

For immunofluorescence analysis HUVEC were cultivated on glass coverslips after coating with 5 µg/ml fibronectin (Cat# 11051407001, Merck, Roche) in PBS. HUVEC were fixed with Roti Histofix (Carl Roth) containing 4% formaldehyde or 4% formaldehyde in PBS. After blocking with normal serum (5% v/v) from the same species as the secondary antibody and permeabilization with 0.3% Triton X-100 in PBS the respective primary antibodies were added and incubated for 60 min at 37 °C or overnight at 4 °C. This was followed by incubation with secondary antibodies donkey IgG anti-mouse IgG (H + L)-Cy3 and donkey IgG anti-rabbit IgG (H + L)-Alexa Fluor 488 (Dianova) at 37 °C for 30 min or 120 min at room temperature. After antibody incubation steps, cytoskeletal F-actin was stained with Alexa Fluor 488 or Alexa Fluor 647 phalloidin (#A12379 and #A22287, Invitrogen, Thermo Fisher Scientific) at a 1:40 and 1:100 dilution, respectively, and with Alexa Fluor 488-coupled anti ZO-1 antibody at a 1:100 dilution. Nuclei were counterstained during mounting with Fluoromount-G Mounting Medium with DAPI (Invitrogen, Thermo Fisher Scientific) and analyzed on Olympus IX73 fluorescent microscope, Leica TCS-SP8 confocal microscope or Zeiss AxioObserver epifluorescent equipped with ApoTome.2 module. All analyses of immunofluorescence images were performed using ImageJ software. Minor adjustments of brightness and contrast were carried out with CorelDRAW 2020 software.

### siRNA transfection

Following siRNA reagents were obtained from Dharmacon: ON-TARGETplus Human RHOA siRNA SMARTpool (#L-008395–00–0005, Dharmacon) containing a mixture of RhoA targeting sequences: CGACAGCCCUGAUAGUUUA, GACCAAAGAUGGAGUGAGA, GCAGAGAUAUGGCAAACAG and GGAAUGAUGAGCACACAAG; ON-TARGETplus Human RHOB siRNA SMARTpool (#L-008395–00–0005, Dharmacon) containing a mixture of RhoB targeting sequences: GCAUCCAAGCCUACGACUA, CAGAACGGCUGCAUCAACU, CGACGAGCAUGUCCGCACA and AAGCACUUCUGUCCCAAUUG. siRNA SMARTpools targeting RhoA and RhoB were applied separately or in combination to deplete RhoA or RhoB. As a control, ON-TARGETplus Non-targeting Control Pool siRNA (#D-001810–10-05, Dharmacon) was used. HUVECs were seeded on fibronectin-coated (5 µg/ml) coverslips (8 × 10^4^ per well of a 24 well plate) or ECIS arrays (8 × 10^4^ per well) and transfected with DharmaFECT 1 (Dharmacon/GE-Healthcare) 24 h later according to the manufacturer’s protocol. Transfection reaction contained the final concentration of 25 nM of the respective siRNA component and 0.2% of DharmaFECT 1 in OptiMEM medium (applied in a total volume of 300 µl per well of the ECIS plate or 500 µl per well of the 24 well plate) per condition. Transfection medium was replaced after 6 h with maintenance medium.

### ECIS for in vitro assessment of endothelial barrier

Endothelial barrier function was measured with electrical cell-substrate impedance sensing (ECIS) Z Theta device (Applied Biophysics). For ECIS measurements, HUVEC were seeded on fibronectin-coated (5 µg/ml) ECIS 8w10e + arrays containing 40 gold intercalated electrodes (Applied Biophysics). Immediately after seeding, recording of the HUVEC monolayer resistance at 4000 Hz was initiated and continued according to the experimental set-up. siRNA transfection was carried out at 24 h post-seeding or in the case of mock- and RuV-infected HUVEC 24 h after detachment and replating of infected cells at 48 hpi.

### Western blot analysis

For western blot analysis, lysates were generated in RIPA buffer supplemented with phosphatase and protease inhibitors. Protein concentration was determined using Pierce BCA Assay Kit (Thermo Fisher Scientific). Equal amounts of protein were loaded onto SDS–PAGE gels, separated by electrophoresis, transferred to nitrocellulose membranes, and subsequently analyzed by immunoblotting. Protein bands were visualized on ChemiDOC MP Imaging System (BioRad) using Western BLoT Ultra Sensitive HRP Substrate (TaKaRa) or Western HRP Substrate (Immobilon® Forte). Quantification of western blots was performed using ImageJ software.

### Statistical analysis

Statistical analysis was performed using GraphPad Prism software. All graphs represent means ± SD, unless otherwise stated in corresponding figure legends. Unpaired Student’s t test was applied when only two groups of samples were compared. Otherwise, one-way ANOVA with Tukey’s or Dunnett’s post-hoc multiple comparison tests was applied.

## Results

### At late rubella infection stages endothelial cells display moderate loss of viability and an elongated morphology

**Fig. 1:**
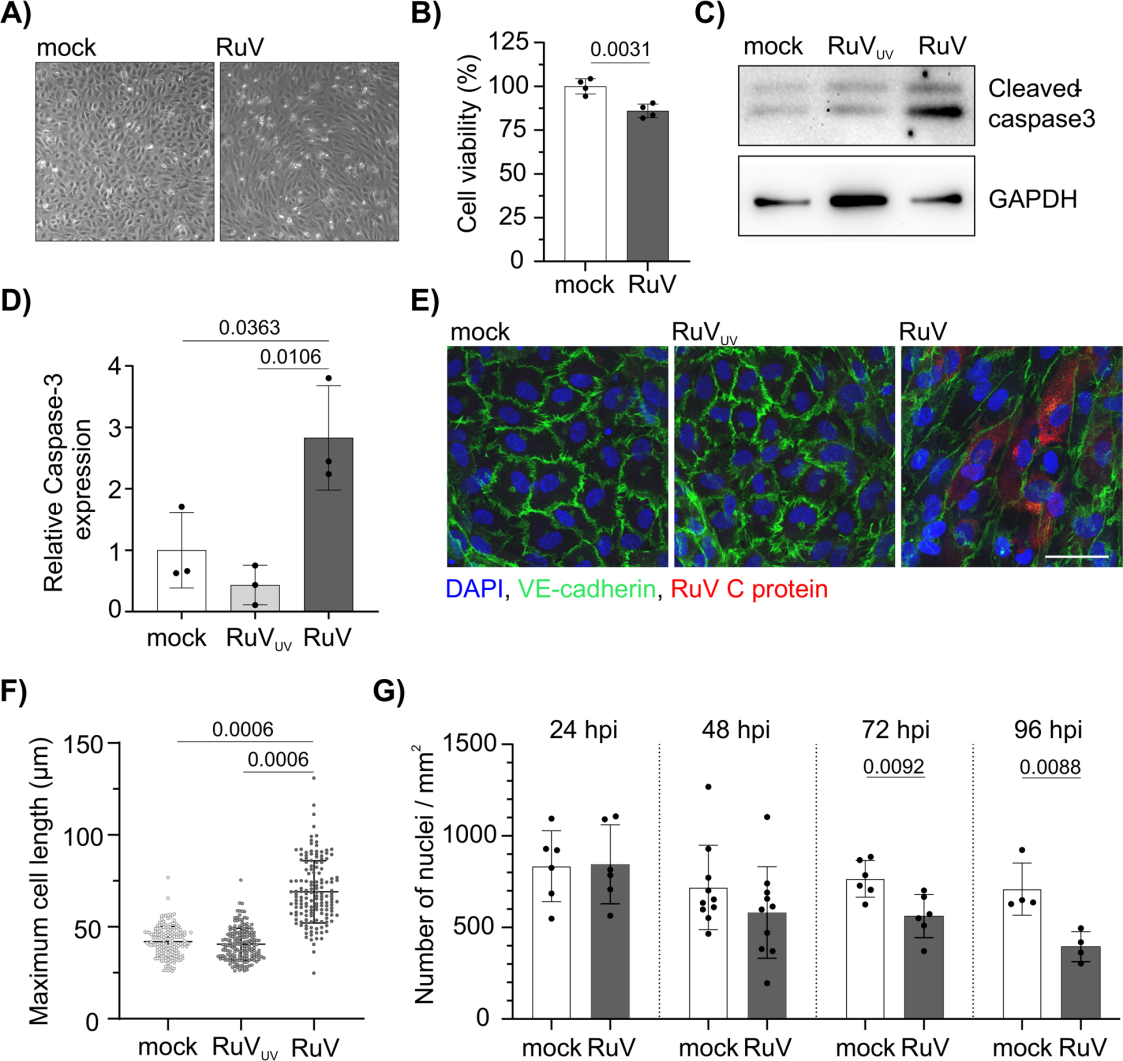
Rubella infection activates caspase-3 and leads to cell elongation in endothelial cells. **A** HUVEC were seeded in 24-well plate and infected with RuV (MOI 10) 3 h later. Bright field microscopy images were obtained at 72 hpi. **B** Cell viability was measured in HUVEC at 72 hpi with RuV using the CCK-8-kit. Cell viability of mock samples was set to 100%. *n* = 4. **C** Western blot analysis of cleaved caspase-3 levels in HUVEC at 72 hpi. GAPDH blot is shown as a loading control. Mock and UV-treated RuV were used as negative controls. **D** Densitometric quantification of the cleaved caspase-3 immunoblots. The graph depicts normalized values relative to the mock control condition. *n* = 3. **E** Immunofluorescence analysis of HUVEC infected with RuV. At 96 hpi the cells were fixed and stained with antibody against viral C protein (red), VE-cadherin (green) and DAPI (blue). The scale bar represents 50 µm. **F** Maximum cell length in HUVEC was measured at 96 hpi 151–172 cells were measured per condition in total. *n* = 3. **G** Nuclei count per mm2 in HUVEC infected with RuV at indicated timepoints. DAPI positive cell nuclei were counted in three separate microscopic images in up to 10 different experiments. *n* = 6 at 24, n=10 at 48, n=6 at 72 and and n=4 at 96 hpi

We followed up on previous reports of cytopathic effects (CPE) and changes in mechanical properties in endothelial cells upon RuV Infection [9, 14]. RuV infection in primary human umbilical vein endothelial cells (HUVECs) resulted at 72 h post-infection (hpi) in slight cell detachment and notably, in an elongated morphology across the cell monolayer with slight variations among individual cells (Fig. 1A).

At this late infection stage CPE development was accompanied by a slight, but significant (14,02% ± 2,94%) loss of viability compared to the mock control (Fig. 1B). In comparison to the controls, UV-inactivated RuV (RuV_UV_) and mock (uninfected) controls, apoptosis induction was evident through significant activation of caspase-3 at 72 hpi (Fig. 1C and D). Immunofluorescence analysis of the cell adhesion protein vascular endothelium (VE)-cadherin at 96 hpi visualized the elongated cell morphology during RuV infection, which was notable in both viral antigen-positive and -negative cells. (Fig. 1E). Measurement of maximum cell length by ImageJ software confirmed a significant increase in cell length distribution after RuV infection in comparison to the controls, RuV_UV_ and mock infection (Fig. 1F). In line with the hypothesis on the impact of an elongated cell size on cell growth area, the number of nuclei per microscopic image was decreased (Fig. 1G). In summary, RuV infection altered endothelial morphology, promoting an elongated appearance within the monolayer.

### Rubella infection disrupts endothelial barrier integrity at late infection stages

**Fig. 2:**
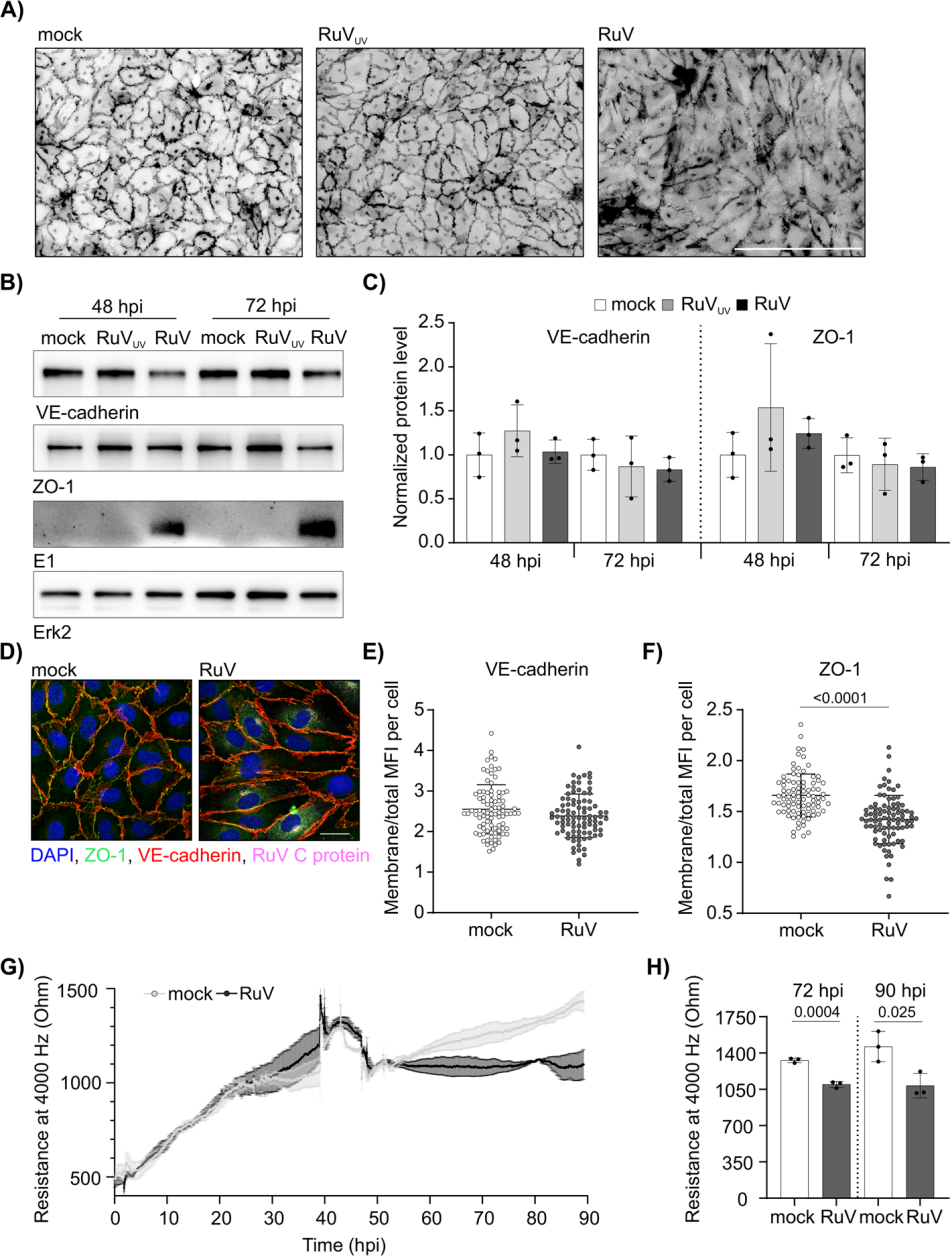
Endothelial barrier integrity is impaired at late stages of rubella infection. **A** Immunofluorescence staining of VE-cadherin in infected HUVEC at 96 hpi. The inverted VE-cadherin signal is shown for better representation of the cell–cell junction disruption. The scale bar represents 200 µm. **B** Western blot analysis of VE-cadherin, ZO-1 and RuV E1 levels in HUVEC at 48 and 72 hpi. Mock and UV-treated RuV were used as negative controls. Erk2 blot is shown as a loading control. **C** Densitometric quantification of the VE-cadherin and ZO-1 immunoblots. The graph depicts normalized values relative to the mock control conditions. *n* = 3. **D** Immunofluorescence analysis of ZO-1 and VE-cadherin together with RuV C protein at 72 hpi. ImageJ-based analysis of VE-cadherin **E** and **F** ZO-1 distribution as the ratio of membrane to total MFI per cells. Membrane area was defined as a 3 µm thick line. *n* = 3, 90 cells per condition in total. **G** ECIS measurement of endothelial barrier integrity in RuV infected HUVEC. The cells were infected at 2 h post-seeding and the resistance at 4000 Hz was recorded until 90 hpi. Mock treatment was used as a negative control. Error bars represent SEM from 3 independent experiments. **H** Quantification of the endothelial monolayer resistance measured at 4000 Hz at timepoints 72 hpi and 90 hpi

The elongated morphology noted for RuV infection could be indicative for alterations in cell–cell junction formation and stability of the endothelial cell monolayer. To visualize monolayer integrity, low-magnification immunofluorescence images of VE-cadherin expression were converted to 8-bit format and inverted. Figure 2A shows a representative example of the resulting inverted fluorescence images with a densely packed monolayer in mock- and RuV_UV_-treated HUVECs opposing extensive gap formation between the dark-colored cell boundaries (VE-cadherin-positive areas) in RuV-infected cells indicative for an altered endothelial cell monolayer. However, western blot analysis revealed no changes in expression of VE-cadherin and zonula occludens-1 (ZO-1) as representative components of cell–cell junctions (Fig. 2B and C). Up to this point no differences were noted between mock- and RuV_UV_-infected

HUVECs, such that we hereafter focused on the comparison between mock and RuV infection. As a follow-up on these observations, immunofluorescence analysis of VE-cadherin and ZO-1 was performed (Fig. 2D). To study cellular distribution of these proteins the ratio of mean fluorescence intensity (MFI) at the cell membrane to whole cell MFI was calculated. While the MFI ratio of VE-cadherin staining was comparable to the control (Fig. 2E), membrane association of ZO-1 was significantly reduced after infection (Fig. 2F). As a next step, we used ECIS to measure endothelial barrier integrity during RuV infection. Here, the measurement of the resistance against a preset flow of electrical current reflects the stability of the cell–cell junctions and as such of the barrier function. At late stages of infection resistance decreased as exemplified in Fig. 2G. While resistance was continuously increasing in mock-infected cells over time of cultivation, the resistance in RuV-infected HUVEC remained at a constant level. This impairment in endothelial barrier integrity resulted in a significant reduction of resistance values in RuV-infected cells measured at 72 hpi (1096.7 Ω vs 1329.61 Ω in mock condition) and 90 hpi (1085.64 Ω vs 1462.25 Ω in mock condition), (Fig. 2H). In summary, we found that RuV induced a significant reduction in endothelial resistance by up to 25.8% compared to the mock control. This demonstrates that RuV infection is associated with a loss of endothelial barrier integrity.

### Actin cytoskeleton reorganization and elevated RhoB levels occur over time of infection

**Fig. 3:**
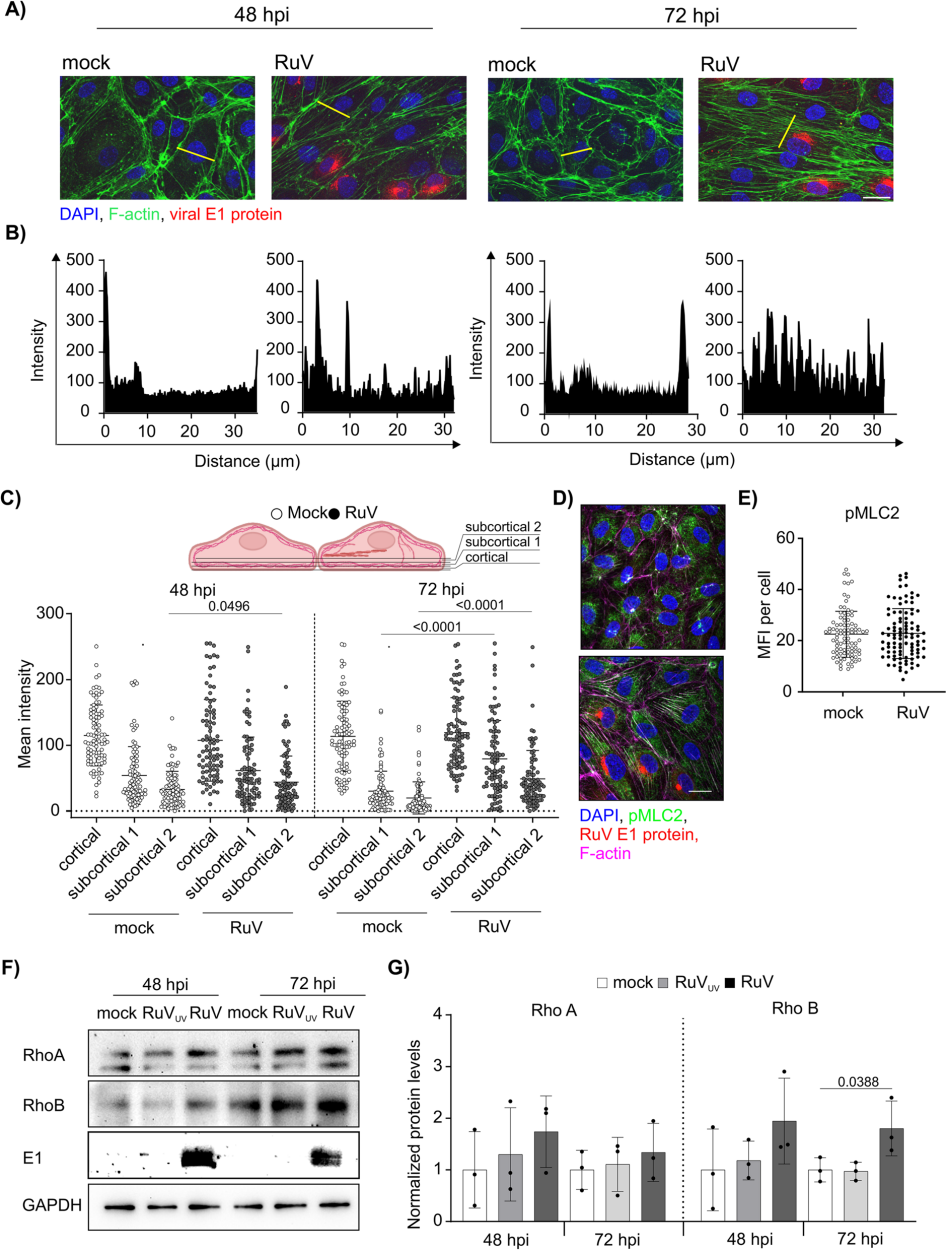
Rubella virus infection increases stress fiber formation and RhoB expression. **A** Labeling of actin cytoskeleton with Alexa Fluor 488 phalloidin (green) together with immunofluorescence staining of viral E1 protein (red) and staining of nuclei with DAPI (blue) in HUVEC at 48 and 72 hpi. The scale bar represents 25 µm. **B** Line plots of phalloidin fluorescence intensities. Fluorescence intensity was measured along the lines marked in yellow in panel A) using ImageJ software. **C** Distribution of actin fluorescence intensities along the line plots at 48 hpi and 72 hpi. Quantification is shown for cellular regions as defined in the graphical scheme (cortical 0 to 1.1 µm; subcortical 1 1.2 to 2.7 µm and subcortical 2 2.8 to 4.4 µm). *n* = 3, 90 cells per condition in total. **D** Immunofluorescence staining of pMLC2 in infected HUVEC at 96 hpi. Scale bar 20 µm. **E** Mean fluorescence intensity of the pMLC2 staining as representatively shown in (D) was quantified in 92–96 cells per condition in total using ImageJ. n = 3. **F** Western blot analysis of RhoA, RhoB and RuV E1 levels in HUVEC at 48 and 72 hpi. Mock and UV-treated RuV were used as negative controls. GAPDH blot is shown as a loading control. **G** Densitometric quantification of RhoA and RhoB immunoblots. The graph depicts normalized values relative to mock. *n* = 3

The integrity of the endothelial monolayer is maintained by cell–cell adhesion complexes, which are regulated by the dynamics of the actin cytoskeleton network. As a next step, content and distribution of actin filaments (F-actin) were analyzed through staining with Alexa Fluor 488 phalloidin. Over time of infection, the number of intracellular stress fibers increased from 48 to 72 hpi in comparison to the mock control (Fig. 3A). Next, we used line plots to visualize the cellular F-actin distribution in the images exemplarily shown in Fig. 3A. Here, we analyzed distribution of phalloidin fluorescent signal along the line drawn across the cell body for one representative cell in each image. The line profile analysis shown in Fig. 3B reveals an altered distribution of F-actin intensity throughout the cell upon RuV infection, while no change was detected over time of incubation for mock infection. Irrespective of mock or RuV infection, high peaks were noted at the beginning and end of the line plots. This reflects actin bundles at cell–cell boundaries which appeared not to be altered upon RuV infection. To quantify the observed redistribution of F-actin from the periphery towards the cell interior, we defined three areas as schematically indicated in Fig. 3C: cortical (periphery to 1.1. µm) and subcortical 1 and 2 (1.2 µm to 2.7 µm and 2.8 µm to 4.4 µm, respectively). A significant increase in F-actin intensity was detected in subcortical region 2 at 48 hpi (Fig. 3C). This effect was more pronounced at 72 hpi where RuV infected cells showed increased F-actin localization in both subcortical areas 1 and 2 in comparison to mock-infected cells. This enhanced actin stress fiber formation coincided with a redistribution of phosphorylated myosin light chain 2 (pMCL2), as a regulator involved in their formation and contractility [15], to these structures (Fig. 3D). Despite its translocation during infection, the MFI was comparable to the uninfected control (Fig. 3E). Next, we examined the expression of Rho GTPases A and B, which in addition to the downstream effector pMLC2, are key regulators of actin dynamics, including stress fiber formation and de novo actin polymerization [16]. As shown in Fig. 3F and G, only RhoB protein levels were significantly increased at 72 hpi. In conclusion, RuV infection was accompanied by enhanced formation of actin stress fibers in subcortical regions of the cell at later time points of infection.

### Entry and early post-entry steps of rubella infection are supported by Rho GTPases

**Fig. 4:**
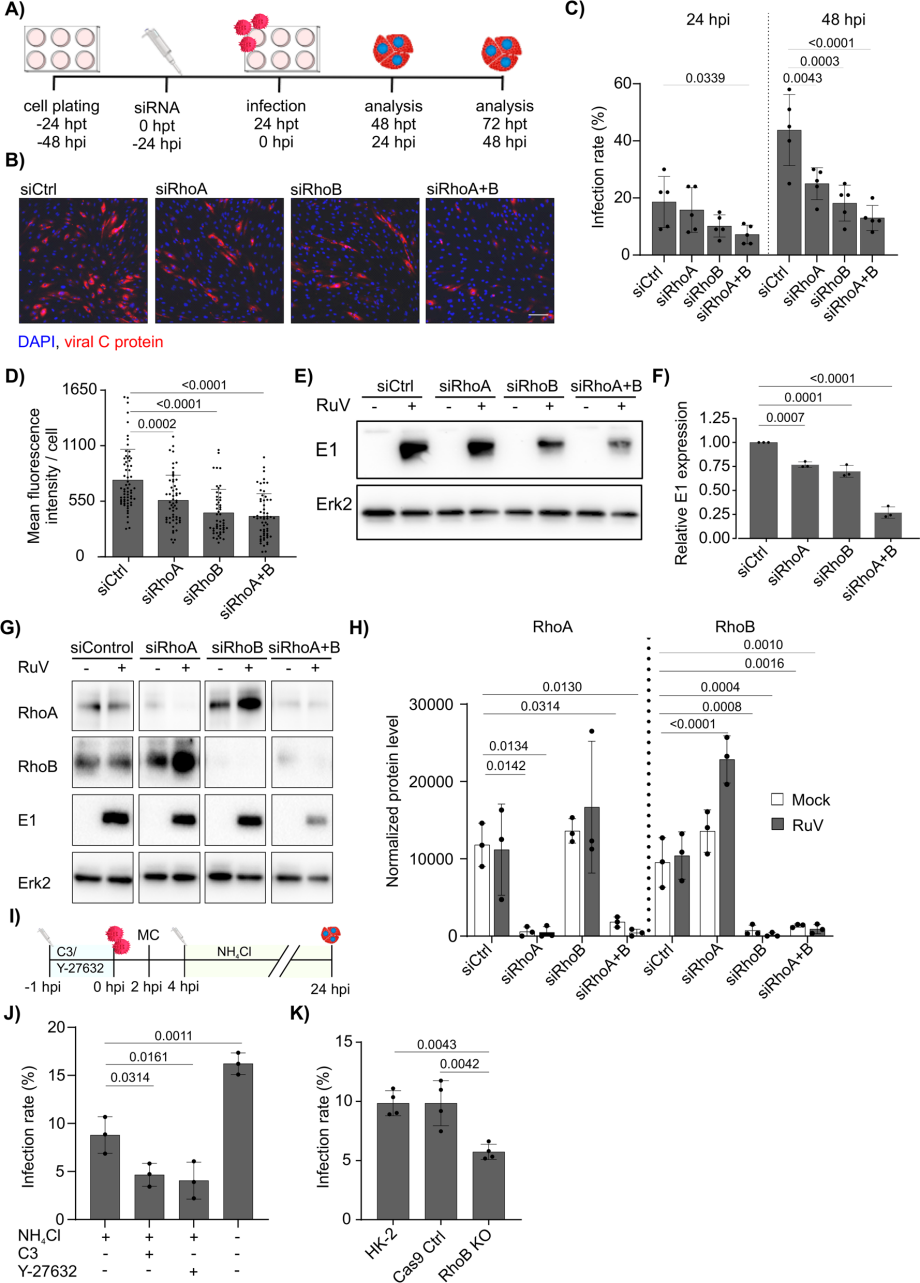
Depletion of RhoA and RhoB strongly decrease rubella infection rate in endothelial cells. **A** Experimental scheme for analyzing the effects of RhoA and RhoB depletion on RuV infection rate. **B** Immunofluorescence staining of viral C protein in RhoA and RhoB single and double KD HUVEC at 48 hpi. The cells were transfected with siRNA control or siRNAs targeting RhoA and RhoB and infected with RuV according to A). At 48 hpi the cells were fixed and stained with antibody against RuV capsid protein (red) and DAPI (blue). The scale bar represents 100 µm. **C** The infection rate was calculated as number of RuV C protein positive cells divided by total cell number per image. 5 separate images were analyzed per condition. For 48 hpi *n* = 5 and for 72 hpi *n* = 6. **D** Quantification of the mean fluorescence intensity of the RuV C protein per cell at 48 hpi. *n* = 3, 50–63 cells per condition. **E** Western blot analysis of the RuV E1 levels in RhoA and RhoB KD HUVEC at 48 hpi. Erk2 blot is shown as a loading control. **F** Densitometric quantification of E1 expression, normalized to siCtrl treatment. *n* = 3. **G** Western blot analysis of the RhoA and RhoB levels in HUVEC at 48 hpi. Erk2 blot is shown as a loading control. **H** Densitometric quantification of the RhoA and RhoB immunoblots in RhoA and RhoB and RhoA/B double KD HUVEC. *n* = 3. **I** Experimental scheme of the application of inhibitors of Rho/ROCK signaling pathway followed by the weak base (lysosomotropic) NH_4_Cl. **J** Impact of NH_4_Cl on RuV infection rate as single or combined treatment with Rho/ROCK inhibitors. 6 separate images were analyzed per condition. *n* = 3. **K** RuV infection rate in RhoB KO kidney tubular epithelial cell line HK-2 compared to HK-2 maternal cell line and Cas9 ctrl cells, *n* = 3

Given the significant upregulation of RhoB during RuV infection, we further investigated its functional role by siRNA-mediated knockdown (KD). Notably, RhoB can affect cell–cell junction protein through enhanced ROCK signaling [11]. We performed single and double KDs of RhoB together with RhoA as they cross-regulate their expression levels [12]. As indicated in the experimental scheme shown in Fig. 4A, HUVEC were mock- and RuV-infected at 24 h post-(siRNA) transfection (hpt) with RuV and the infection rate was determined at 24 hpi (48 hpt) and 48 hpi (72 hpt) using immunofluorescence staining of viral C protein and counting by ImageJ software. As shown in representative immunofluorescence images (Fig. 4B), depletion of RhoA or RhoB decreased the number of rubella-positive HUVEC. More detailed analysis revealed that in comparison to depletion of RhoA a more pronounced reduction in infection rate was noted after the depletion of RhoB. At 48 hpi all KD conditions reduced the infection rate significantly in comparison to the control siRNA condition (Fig. 4C). The strongest effects were observed in double RhoA/RhoB KD cells, where rubella infected only 13.0% of cells in comparison to 43.7% cells in control KD condition at 48 hpi. These data suggest that RhoA and RhoB are not functionally redundant during rubella infection in endothelial cells. Although the number of infected cells increased from 24 to 48 hpi following knockdown of RhoA and RhoB, the extent of this increase was reduced compared to the siControl (siCtrl) condition. Subsequent analysis of the staining intensity of RuV C protein as a representative viral protein revealed that depletion of RhoA or RhoB also significantly decreased its expression level per cell (Fig. 4D). Also in this analysis, the loss of RhoB had stronger impact in comparison to RhoA. To complement our immunofluorescence analysis, we investigated rubella E1 protein expression at 48 hpi (72 hpt) in HUVEC depleted of RhoA, RhoB or RhoA/RhoB combined by western blot analysis. As shown in Fig. 4E and F, viral E1 protein levels were significantly reduced in cells depleted of RhoA or RhoB. E1 levels dropped to only 27% in double RhoA/RhoB KD in comparison to siCtrl condition. Moreover, western blot analysis confirmed efficient reduction of RhoA and RhoB levels in cells transfected with respective siRNAs and in double KD HUVEC (Fig. 4G). Interestingly, RhoB levels were strongly elevated upon rubella infection only in RhoA knockdown cells, whereas no significant upregulation of RhoB was observed in mock-infected cells at 48 hpt (Fig. 4H). In addition to siRNA-mediated knockdown, we applied *Clostridium botulinum* exoenzyme C3 transferase (C3), which inhibits GTPases RhoA, RhoB and RhoC. Alternatively, to inhibit the downstream ROCK signaling we used the ROCK inhibitor Y-27632. The addition of these pharmacological compounds prior to infection enabled a more detailed distinction of the contributory role of Rho GTPases during viral entry versus later steps of the virus life cycle. As summarized in the experimental scheme shown in Fig. 4I, we combined this approach with the application of the lysosomotropic weak base NH_4_Cl at 20 mM. NH_4_Cl is known to inhibit viruses including RuV at early stages through its ability to neutralize acidic cellular compartments and as such to interfere with fusion and uncoating in the endosomal pathway [17, 18]. Treatment of HUVEC with C3 or Y-27632 1 h prior to infection followed by wash-out and addition of NH_4_Cl at 4 hpi significantly reduced the number of RuV-infected cells in comparison to NH_4_Cl alone (Fig. 4J). Addition of NH_4_Cl at 4 hpi reduced the number of infected cells significantly in comparison to the untreated control. Based on the non-redundant role of RhoA and RhoB and the upregulated RhoB expression during RuV infection, we infected the RhoB KO kidney tubular epithelial cell line HK-2 to test whether these observations were specific for endothelial cells. RhoB KO cells showed a reduction in infection rate of 42% at 48 hpi in comparison to the maternal cell line or the control Cas9 cells (Fig. 4K). Although the infection rates in HK-2 cells were fourfold lower in comparison to HUVEC, the reduction in infection rate in RhoB KO cells was comparable to siRhoB-transfected HUVEC (Fig. 4C). In conclusion, depletion of RhoA and even more prominently of RhoB significantly impaired the efficiency and progression of RuV infection at early post-entry steps through a non-cell type-specific mechanism.

### *Supernatant of rubella-infected endothelial cells promotes cell elongation *via* CCL5 in RhoA/B-independent manner*

**Fig. 5:**
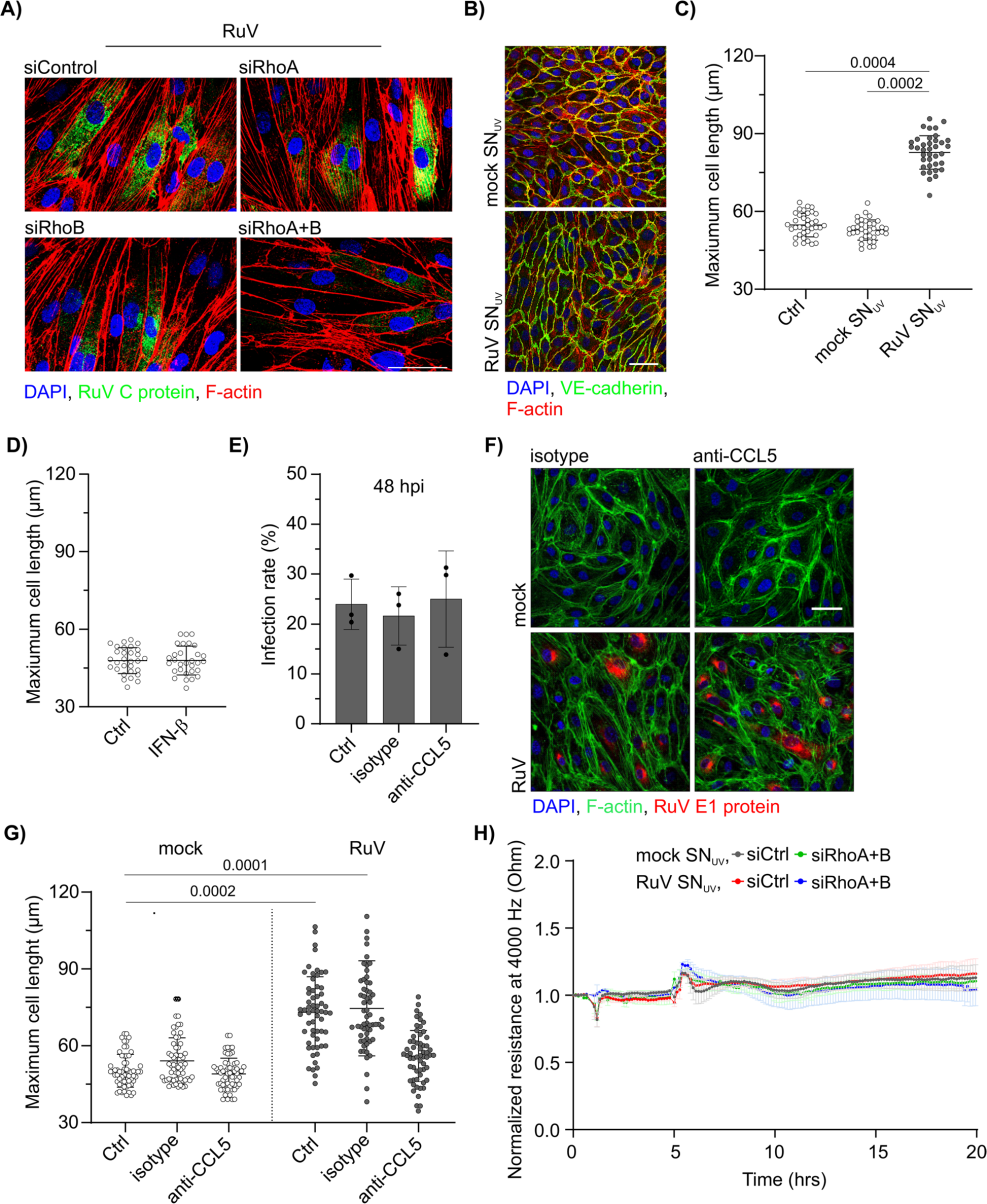
CCL5 chemokine is required for rubella-induced endothelial cell elongation. **A** Staining of F-actin with Alexa Fluor phalloidin 488 (red) together with immunofluorescence staining of RuV C protein (green) in RhoA and RhoB KD HUVEC at 48 hpi together with labeling of nuclei with DAPI (blue). The scale bar represents 50 µm. **B** Immunofluorescence staining of HUVEC treated with the UV-inactivated supernatant obtained from mock- and RuV-infected HUVEC collected at 72 hpi. Cells were treated with the supernatant for 4 h and fixed for staining after 48 h of incubation. VE-cadherin (green), phalloidin (red) and DAPI (blue). Scale bar represents 50 µm. **C** Maximum cell length in HUVEC at 48 h after addition of the supernatant. 36 cells, *n* = 3. **D** Maximum cell length in HUVEC treated with IFN-β (10 ng/ml) for 48 h. 30 cells, *n* = 3. **E** The RuV infection rate in HUVEC in presence of the anti-CCL5 antibody or isotype control, *n* = 3. **F** Immunofluorescence staining of HUVEC infected with RuV and treated with the anti-CCL5 antibody CCL5 antibody or isotype control added at 0.1 µg/ml at 8 and 20 hpi. F-actin was labeled with Alexa 488 phalloidin (green), RuV E1 (red), and DAPI for nuclei (blue). Scale bar represents 50 µm. **G** Maximum cell length in HUVEC at 48 h after addition of the CCL5-depleted supernatant. 46–60 cells, *n* = 3. **H** Endothelial barrier integrity in siCtrl and siRhoA + B transfected HUVEC treated with the UV-inactivated supernatant obtained from mock- and RuV-infected HUVEC collected at 72 hpi. Endothelial resistance was measured using ECIS at 4000 Hz for 20 h. Resistance values normalized to 1 h prior to treatment are shown. Error bars represent SEM. *n* = 3

Next, we analyzed the influence of RhoA and RhoB on actin cytoskeleton morphology in RuV-infected HUVEC. As shown in Fig. 5A, the prominent elongated morphology was present in all conditions. This indicates that RuV infection-associated cell elongation affected the entire monolayer irrespective of RhoA, RhoB or viral antigen expression or overall infection rate. Thus, we next investigated if RuV-infected cells secrete a soluble factor that might initiate endothelial cell elongation independent from viral infectious cycle. For this purpose, we inactivated infectious virus in the supernatant through exposure to UV light. As shown in Fig. 5B, UV-inactivated supernatant from RuV-, but not mock-infected cells induced significant elongation in HUVEC in comparison to the untreated control. Quantification of maximum cell length confirmed the significance of this observation and the contributory role of a secreted factor (Fig. 5C). In our previous study, we found that RuV infection in HUVEC induces different cytokines and chemokines including type I interferon (IFN)-β as a major inducer of other soluble factors [1]. However, as shown in Fig. 5D IFN-β treatment was not sufficient to induce cell elongation. Among the previously identified cytokines that were induced by RuV infection, but not by IFN-β treatment, was the chemokine CCL5 [1]. Thus, we next applied neutralizing CCL5-specific antibody to mock- and RuV-infected HUVEC at 72 hpi. Although application of the CCL5 antibody did not alter infection rate (Fig. 5E), it restored cell length to the mock control levels (Figs. 5F and G). Hereafter, we investigated whether the association of these soluble factors such as CCL5 with cell elongation was sufficient to disrupt endothelial monolayer integrity. As shown in Fig. 5H, treating HUVECs with UV-inactivated supernatant collected from RuV-infected cells did not induce endothelial barrier disruption, neither in the siCtrl nor in the siRhoA/B transfected cells.

In conclusion, among the soluble factors secreted by RuV-infected cells, the CCL5 chemokine specifically mediated endothelial cell elongation. Furthermore, this morphological change was not a prerequisite for the destabilization of the endothelial barrier.

### RhoB is involved in stress fiber formation and barrier dysfunction during infection

**Fig. 6:**
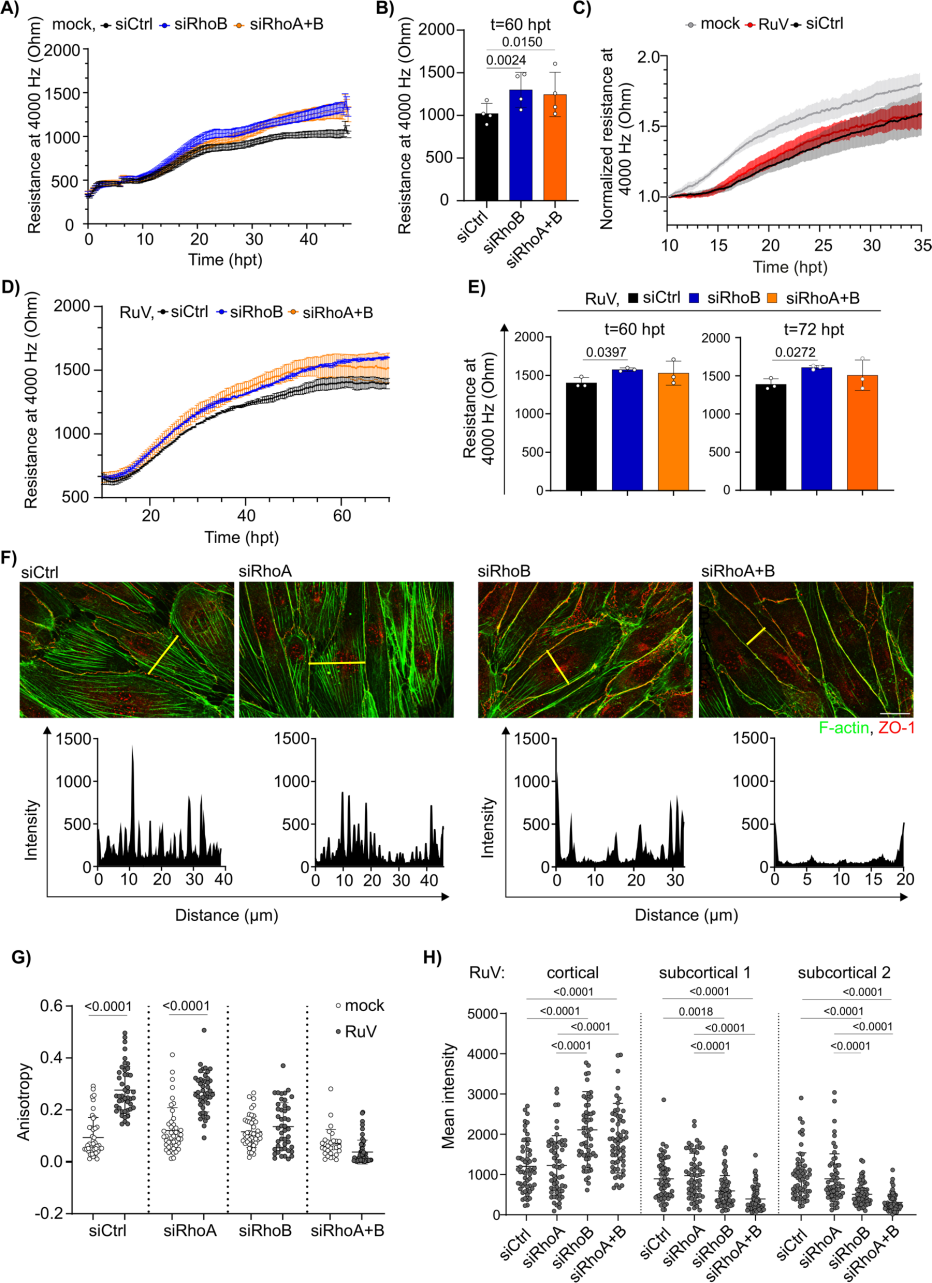
RhoB depletion prevents loss of barrier integrity in association with rubella infection-induced actin rearrangement. **A** Effect of RhoB and RhoA + B depletion on endothelial barrier integrity in HUVEC. Absolute endothelial resistance at 4000 Hz was measured by ECIS in HUVEC transfected with the control siRNA, or siRNAs targeting RhoB or RhoA + B. Absolute values are shown starting at 0 h post siRNA transfection (hpt). Error bars represent SEM. n = 3. **B** Quantification of the endothelial monolayer resistance measured at timepoint 60 hpt. Error bars represent SEM. *n* = 4. **C** Quantification of the endothelial monolayer resistance after normalization to the timepoint 10 hpt. Shown are mock- and RuV-infected HUVEC in comparison to RuV-Infected HUVEC that were detached and newly plated at 48 hpi followed by transfection with siCtrl 24 h later, *n* = 3. **D** Effect of RhoB depletion on endothelial barrier integrity in RuV–infected HUVEC. Endothelial resistance at 4000 Hz was measured by ECIS in RuV infected HUVEC transfected with the siControl, siRhoB or siRhoA + B at 48 hpi. Absolute values are shown starting at 10 hpt. **E** Quantification of the endothelial monolayer resistance measured at 4000 Hz at timepoints 60 hpt and 72 hpt. *n* = 3. **F** Immunofluorescence staining of RhoA and RhoB KD HUVEC infected with RuV at 48 hpi. 24 h prior to RuV infection the cells were transfected with depicted siRNAs. F-actin was labeled with Alexa fluor 488 phalloidin (green) and immunostained with antibody against ZO-1 (red). Images were obtained using the Zeiss AxioObserver. Scale bar represents 25 µm. Line plots of phalloidin intensities measured along yellow lines were obtained using ImageJ software. **G** Anisotropy of the F-actin staining in mock and RuV-infected control, RhoA and RhoB knockdown was measured using the Fiji Plugin FibrilTool at 48 hpi. 30–57 cells, *n* = 3. **H** Distribution of actin fluorescence intensities along the line plots was analyzed as in Fig. 3 C. *n* = 3, 58–62 cells per condition in total

Among the pleiotropic alterations in RuV-infected HUVEC RhoB appears to play an essential role. Hereafter we investigated how these changes together with RhoB expression might contribute to disruption of endothelial barrier as observed in ECIS measurements (Figs. 2G and H). As shown in Figs. 6A and 6B and supported by literature data [12], at 48 hpt RhoB KD alone or together with RhoA enhanced endothelial barrier in mock-infected HUVEC by 28% ± 8%. Next, we applied ECIS measurements on HUVEC with single RhoB and double RhoA/B KD during RuV infection. We adopted the experimental outline due to the transient nature of siRNA-mediated RhoB KD whose efficiency declines after 72 hpt, a timepoint at which RuV starts to impair endothelial barrier (Fig. 2G). Therefore, we trypsinized RuV-infected HUVEC at 48 hpi followed by siRNA transfection after 24 h of incubation. ECIS measurement revealed a course of barrier impairment similar to HUVEC infected directly with RuV prior to ECIS measurement (Fig. 6C). The experimental scheme was extended to transfection of siRhoB and siRhoA/siRhoB. Both, RhoB KD alone and the combined RhoA/RhoB double KD stabilized endothelial barrier under RuV infection (Fig. 6D). However, this effect was only statistically significant with RhoB single KD (Fig. 6E). Next, we aimed to investigate whether the contribution of RhoB to barrier impairment was connected with stress fiber formation during RuV infection.

Microscopic analysis combined with visualization of F-actin distribution through line plots showed that KD of Rho B, but not of RhoA restored actin morphology as shown through absence of stress fiber formation in comparison to siCtrl (Fig. 6F). Additionally, the increase in anisotropy as a measure of fiber alignment and directional organization reflects enhanced formation of parallel actin stress fibers during RuV infection compared to the mock control. However, after RhoB KD either alone or together with RhoA, the level of anisotropy was restored comparable to the mock control (Fig. 6G). This RhoB KD-dependent effect, which was not noted for RhoA KD, was particularly evident through the significant reduction of actin mean fluorescent intensity in subcortical cell regions (Fig. 6H). In conclusion, RhoB expression is required for endothelial barrier impairment during RuV infection, which parallels loss of stress fiber formation in the absence of RhoB.

### ROCK signaling pathway downstream of RhoB is required for endothelial impairment during rubella infection

**Fig. 7:**
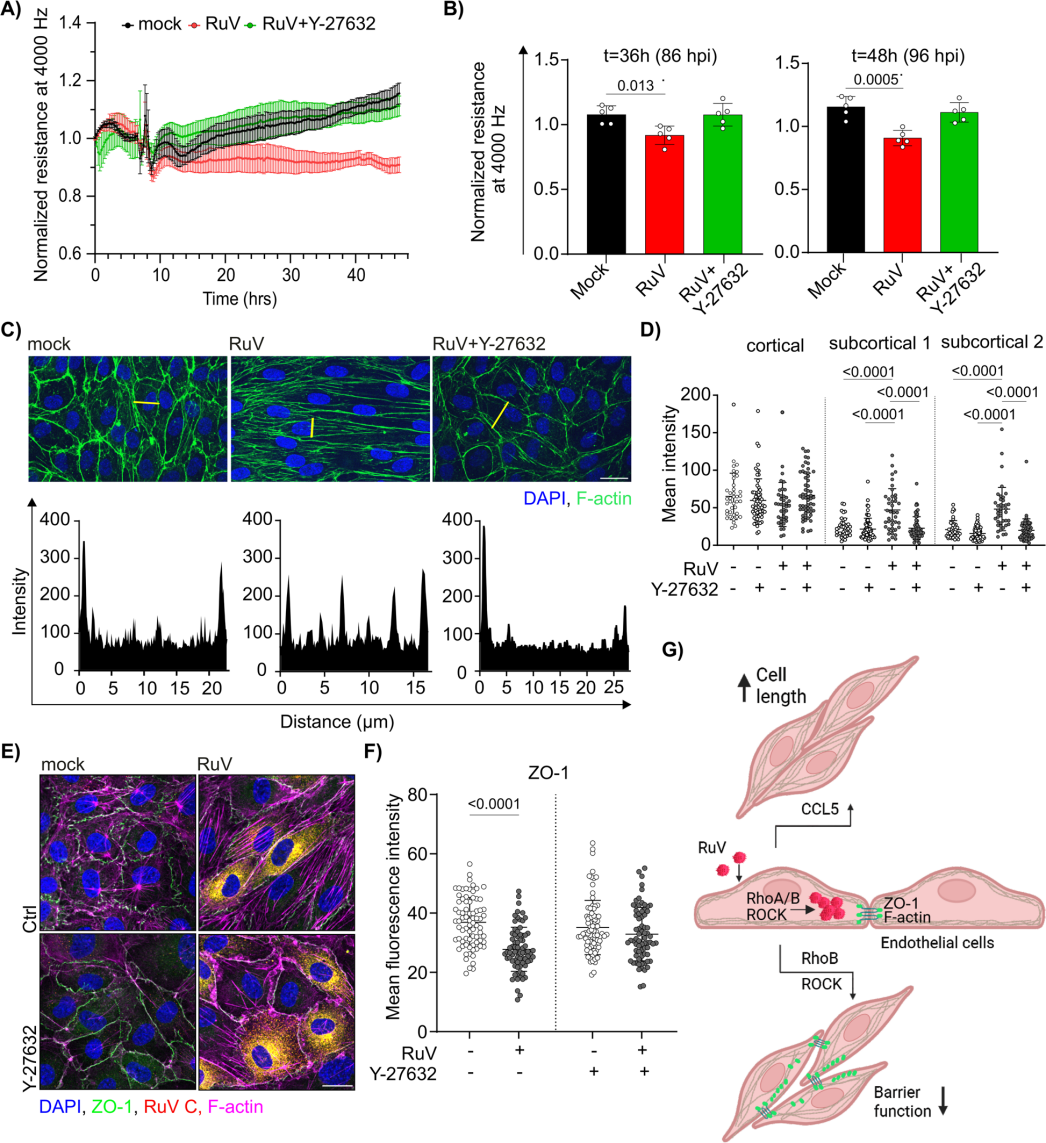
Inhibition of ROCK as downstream pathway of RhoB GTPase was sufficient to prevent loss of barrier integrity during RuV infection. **A** Endothelial resistance at 4000 Hz was measured by ECIS in RuV-infected HUVEC treated at 48 hpi with Y- 27632 (10 μM) for 6 hr. Resistance values normalized to 1 hr prior to treatment are shown. Error bars represent SEM. *n*=5. **B** Quantification of the endothelial monolayer resistance measured at timepoints 36 hr and 48 hr after addition of the inhibitor. *n*=5. **C** Staining of HUVEC infected with RuV and treated with Y-27632 (10 μM) at 48hpi for 6hr for F-actin with Alexa Fluor 488 phalloidin (green) and for nuclei with DAPI (blue). Scale bar represents 25 μm. Line plots of phalloidin intensities are displayed under immunofluorescence images. Fluorescence intensity was measured along the lines marked in yellow using ImageJ software. **D** Distribution of actin fluorescence intensities along the line plots was analyzed as described in the Figure 3 C. *n*=3, 40-65 cells per condition in total. **E** Immunofluorescence staining of ZO-1 in HUVEC treated as in C. RuV C protein antibody was used to demonstrate successful RuV infection and F-actin was stained with Alexa 647 phalloidin. Scale bar, 20 μm. **F** ImageJ-based analysis of the ZO-1 membrane staining. The membrane area was defined as a 3 μm thick line at the cell boundary. *n*=3, 71-83 cells per condition in total. **G** Summarizing figure for the impact of RuV infection on endothelial cells. RhoB and ROCK signaling pathway are central for RuV-induced alteration of endothelial morphology and function. Created in BioRender. Claus, C. (2026) https://BioRender.com/62mrn3t

RhoB does not directly interact with actin but can exert its influence through signaling pathways such as the ROCK pathway. Notably, the addition of Y-27632 ROCK inhibitor at 48 h post-infection for 6 h followed by wash-out restored the resistance values comparable to the mock infection (Fig. 7A). Detailed analysis of endothelial

resistance at 86 and 96 h post-infection showed that treatment of RuV-infected cells with Y-27632 restored resistance values to control levels (Fig. 7B). Next, we aimed to investigate whether ROCK signaling pathway also contributes to the formation of actin stress fibers during RuV infection. Microscopic analysis combined with visualization of F-actin distribution through line plots revealed that addition of Y-27632 to RuV-infected cells restored actin morphology to a pattern similar to mock infected cells (Fig. 7C). Figure 7D confirms restoration of actin distribution in subcortical regions to a level comparable to the uninfected control. In line with the impact of Y-27632 on actin distribution, immunofluorescence analysis of ZO-1 (Fig. 7E) shows recovery of the ZO-1 membrane localization to a level comparable to the uninfected control (Fig. 7F). In summary, the ROCK signaling pathway downstream of RhoB contributes to actin cytoskeleton rearrangement and endothelial barrier disruption during RuV infection. Figure 7G illustrates the role of the RhoB/ROCK signaling axis in driving the RuV-induced changes in barrier integrity and CCL5-secretion-dependent elongated cell morphology.

## Discussion

Endothelial cells are an integral part of rubella pathogenicity including transplacental spread through desquamated endothelial cells [4]. RuV infection in endothelial cells induces only a moderate cytopathic effect through cell rounding and detachment [14] and with only moderate signs of apoptosis at early stage of infection [1]. Here we present that at later stages endothelial impairment becomes evident through morphological alterations up to reduction of barrier integrity. Loss of barrier integrity was rescued by interference with RhoB and ROCK-associated signaling. In resting endothelium, RhoB is a negative regulator of endothelial permeability [11, 12], which is in line with our data presented here. In the context of RuV infection RhoB was upregulated and its knockdown restored endothelial barrier resistance. Cell elongation provides an explanation for the reduction in cell number during infection although global cellular protein synthesis and cell cycle are not affected by RuV infection in HUVEC [19].

Rho GTPases are central in the regulation of cytoskeletal dynamics and as such in viral replication and also in viral pathogenesis [20]. Remodeling of the cytoskeleton network is involved in various aspects of the virus life cycle, including virus entry, replication, assembly and virus spread [21]. A well-studied example in this context is respiratory syncytial virus (RSV) assembly site at the plasma membrane, which involves Rac1 and RhoA-associated pathways and F-actin remodeling through trafficking of Rho GTPases to these sites [22]. These signaling pathways in turn appear to involve activation of Rho GTPases during RSV infection [23]. RhoB as a proviral factor participates in the assembly process of cytomegalovirus in fibroblasts [24]. The Rho/ROCK signaling axis and especially the contribution of RhoA is assigned to different virus species and to various aspects of the viral life cycle, with a rather supporting role [20]. In line with this, the data presented here assign a supporting role especially to RhoB together with ROCK during RuV infection. The application of the lysosomotropic compound NH_4_Cl directly after Rho or ROCK inhibitor application suggests that RuV early post-entry steps are affected. This is in accordance with the role of the Rho/ROCK axis in e.g. receptor clustering and optimizing virus interaction with receptors and coreceptors, intracellular transport of virus particles and rearrangement of actin filaments in support of virus infection [21]. Rho GTPase signaling is also regulating endocytotic processes, which are central to the entry of various viruses [21]. Thus, RhoB effects on endothelial barrier need to be considered in the context of its role during RuV entry and subsequent virus replication. In future studies, the contributory role of the RhoB–ROCK signaling axis in viral genome replication and in the production and release of viral progeny should be addressed. Actin remodeling is not only involved in different steps of the virus life cycle itself, but also in the induction of an immune response during infection and as such in progression of virus infection. Priming of the RIG-I-like receptor (RLR) as a pattern recognition receptor for the induction of an antiviral response involves actin remodeling [25].

Mechanisms contributing to endothelial barrier disruption vary among virus types. While RuV infection reduced ZO-1, but not VE-cadherin cell surface localization without alterations in their expression, Dengue virus infection decreased expression and induced redistribution of both, ZO-1 and VE-cadherin [26, 27]. Likewise, infection of brain microvascular endothelial cells (BMECs) with mouse hepatitis virus type 3 resulted in an increase in permeability together with a reduction in ZO-1 and VE-cadherin expression [28]. In line with our data on RuV, coxsackievirus B3 infection altered actin expression which was followed with a temporal delay by redistribution of ZO-1 [29]. This was suggested to occur through its connection to actin. Our data further show that ROCK-associated pathways contribute to actin stress fiber formation and reduced localization of ZO-1 at the cell–cell junctions. In line with this, the observed changes in pMLC localization during RuV-induced barrier disruption in our experiments are consistent with the study by Hirano & Hirano, which compared mono- and di-phosphorylated MLC in thrombin-treated aortic endothelial cells. Specifically, they observed a biphasic pMLC pattern, characterized by its localization on actin stress fibers during the late phase of barrier disruption [30]. Whereas RhoB signaling is crucial for stress fiber formation, and endothelial barrier disruption, neither RhoA nor RhoB were required for endothelial cell elongation. Our data indicate that among the secreted factors CCL5 chemokine was required for RuV-dependent endothelial cell elongation. This claim is supported by our previous work, where we identified CCL5 as one of the upregulated chemokines in RuV-infected, but not IFN-β-treated HUVECs [1]. Notably, cell elongation induced by UV-inactivated supernatants did not correlate with endothelial barrier disruption.

Despite its high degree of homology to RhoA, RhoB has distinct functions. Due to its localization to endosomes, it regulates endosomal dynamics while also participating in inflammatory responses—particularly in macrophages and endothelial cells—and stress responses, including hypoxia [31–33]. In general, there is more data available for RhoA than for RhoB on its role in virus infections and in maintenance of endothelial barrier function. Although we cannot rule out a contributory role of RhoA, our data identified RhoB as the main contributor of stress fiber formation induced by RuV infection, which is especially noteworthy as this is usually described for RhoA [34]. In HUVEC thrombin-induced RhoA and subsequent ROCK activation results in an increased F-actin stress fiber formation and endothelial permeability [35]. A contributory role was also assigned to RhoB in this process [12]. For Andes virus, which belongs to the pulmonary syndrome-causing hantaviruses, knockdown of RhoA or inhibition of ROCK with fasudil or Y-27632 reduced Andes virus-associated permeability of microvascular endothelial cells [36]. The viral N protein supports release of RhoA from its inhibitor, phosphorylated RhoGDI, and as such promotes activation of RhoA that is induced by hypoxia and VEGF [37].

Our data demonstrate that the RhoB/ROCK signaling axis, with a contribution of MLC2 activation and possibly other unidentified downstream effectors of ROCK, is required for RuV-mediated disruption of endothelial barrier. RhoB also regulates cell migration and the formation of lamellipodial protrusions and could thus be favourable for virus dissemination during in vivo infection [38]. Furthermore, upregulation of RhoB during RuV infection could represent an infection promoting cellular response. Accordingly, treatment of macrophages with several agonists of the toll-like receptor (TLR) family such as lipopolysaccharide and the synthetic dsRNA analogon poly(I:C) induced the expression of RhoB [39]. In this context, RhoB through its interaction with MHCII acts as a positive regulator of TLR-associated antiviral innate immunity signaling [39]. Loss of barrier integrity through the RhoB/ROCK axis could support rubella infection through detachment of endothelial cells from the monolayer and promotion of transendothelial migration of immune cells. Based on the tropism of RuV for lymphocytes [40, 41], these cells could serve as transmigrating virus carriers.

Rho GTPase-ROCK activity is involved in various cellular pathways and regulatory networks including apoptosis [42, 43], inflammation, immune regulation and immune cell migration [44]. In this regard, application of the ROCK inhibitor fasudil in a rat model with collagen-induced arthritis and in rheumatoid arthritis patients-derived cells reduced inflammation and the generation of proinflammatory cytokines [44, 45]. We identified the RhoB-ROCK axis as the main driver of loss of endothelial function during RuV infection exemplified through loss of endothelial barrier integrity. Fasudil is already successfully applied in multiple clinical trials with therapeutic efficacy in inflammatory diseases [44]. Its application in virus infection-associated diseases is beginning to emerge as shown in a mouse model for coxsackievirus-associated myocarditis [46], up to vascular dysfunction in long COVID [47]. As a hypothesis-driven outlook, targeting ROCK signaling e.g. through the clinically approved fasudil could be a potential therapeutic strategy for rubella pathogenesis. Notably, Rho GTPases are involved in cochlear hair cell development [48], which also extends the scope of therapeutic applications to effects during pregnancy.

## Supplementary Information

Supplementary Material 1.

## Data Availability

All data generated or analyzed during this study are included in the article. The datasets used and/or analyzed in this study are available upon request from the corresponding author.
