# Supplementary material for: RhoB supports rubella infection and impairs endothelial barrier integrity through downstream ROCK signaling
Source: Cell Commun Signal. 2026 Apr 27;24:258. doi: 10.1186/s12964-026-02898-w (PMC13126974; doi:10.1186/s12964-026-02898-w)
Supplement: Supplementary file 1 — Supplementary Material 1. [file 12964_2026_2898_MOESM1_ESM.pdf]

Uncropped western blot – Figure 1 C

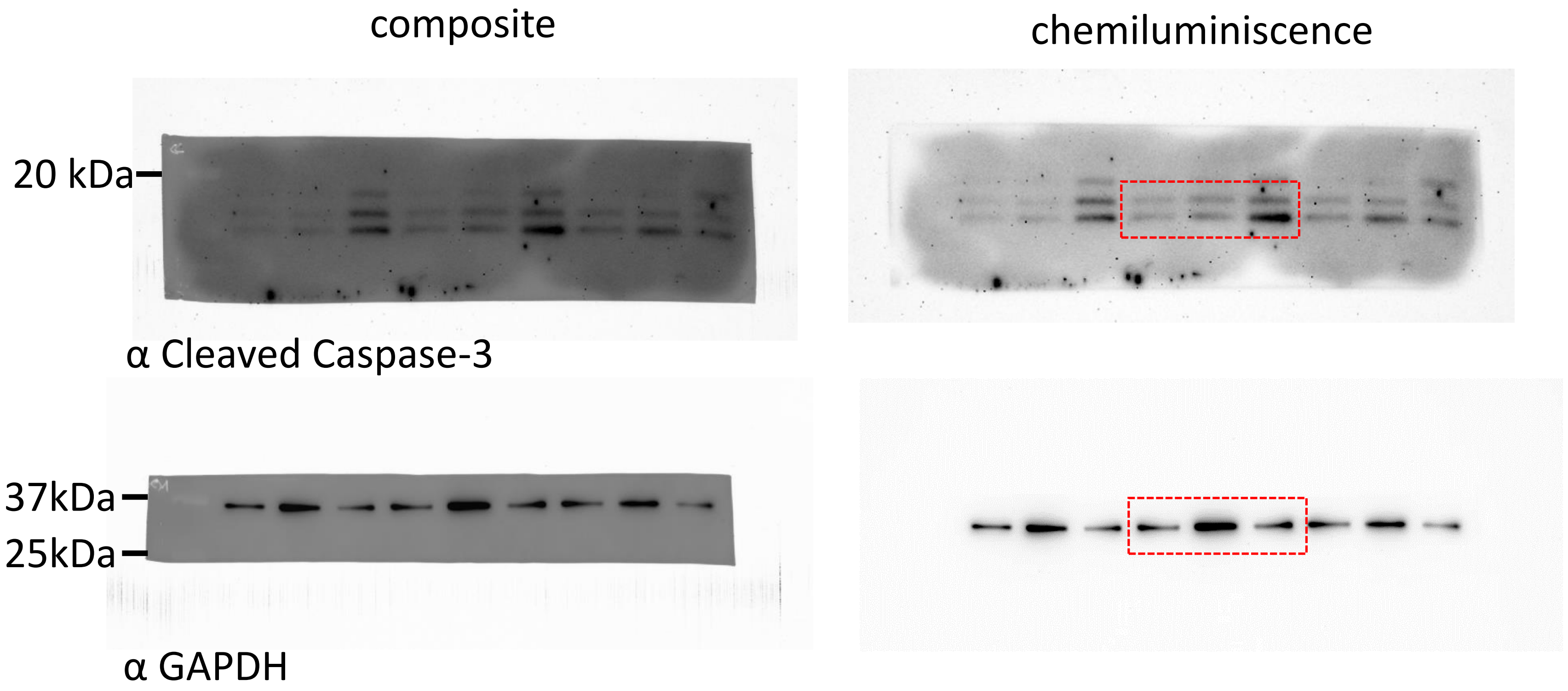

Red rectangles delineate cropped areas shown in the manuscript.

Uncropped western blot – Figure 2 B

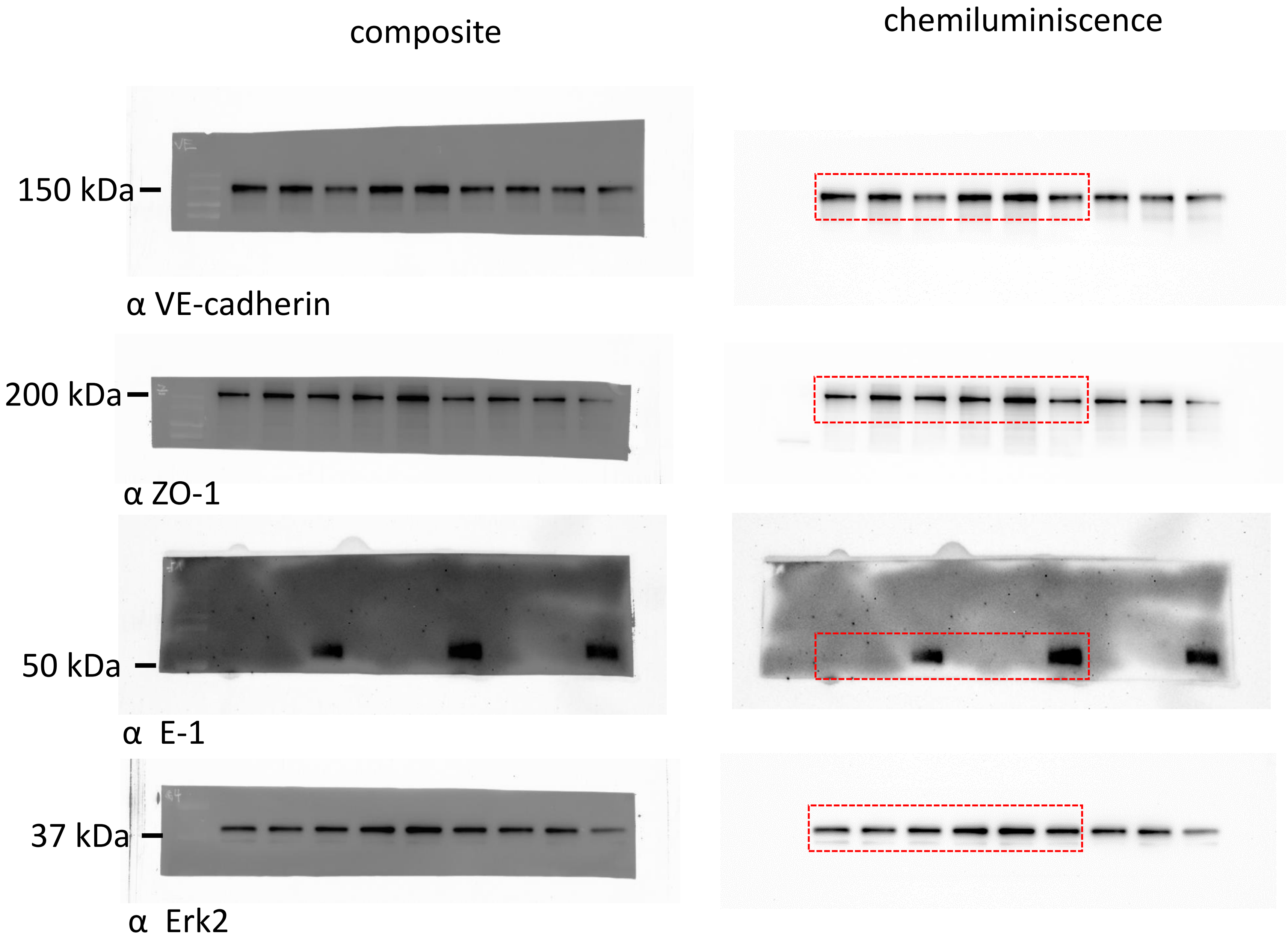

Uncropped western blot – Figure 3 C

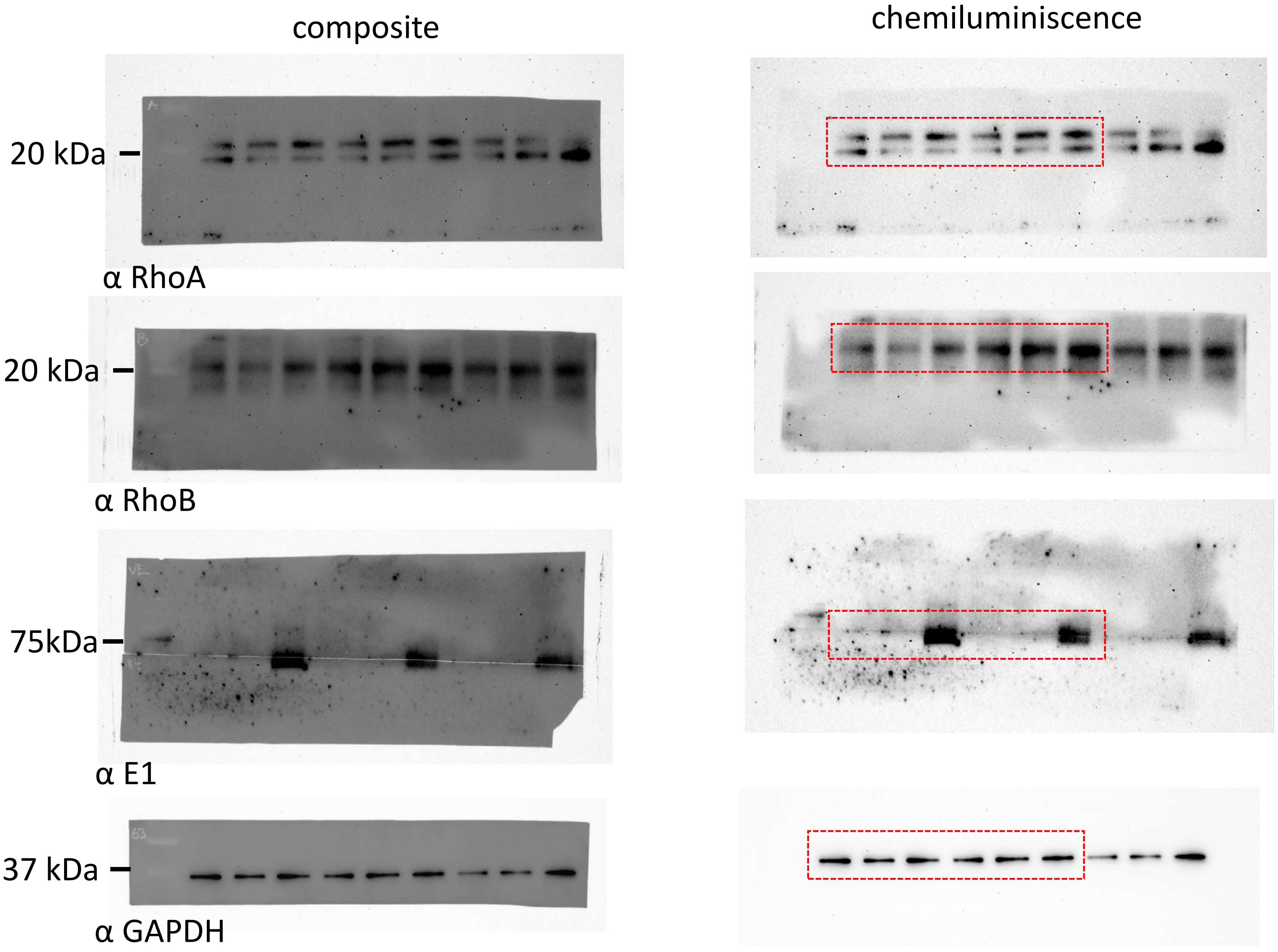

Uncropped western blot – Figure 4E

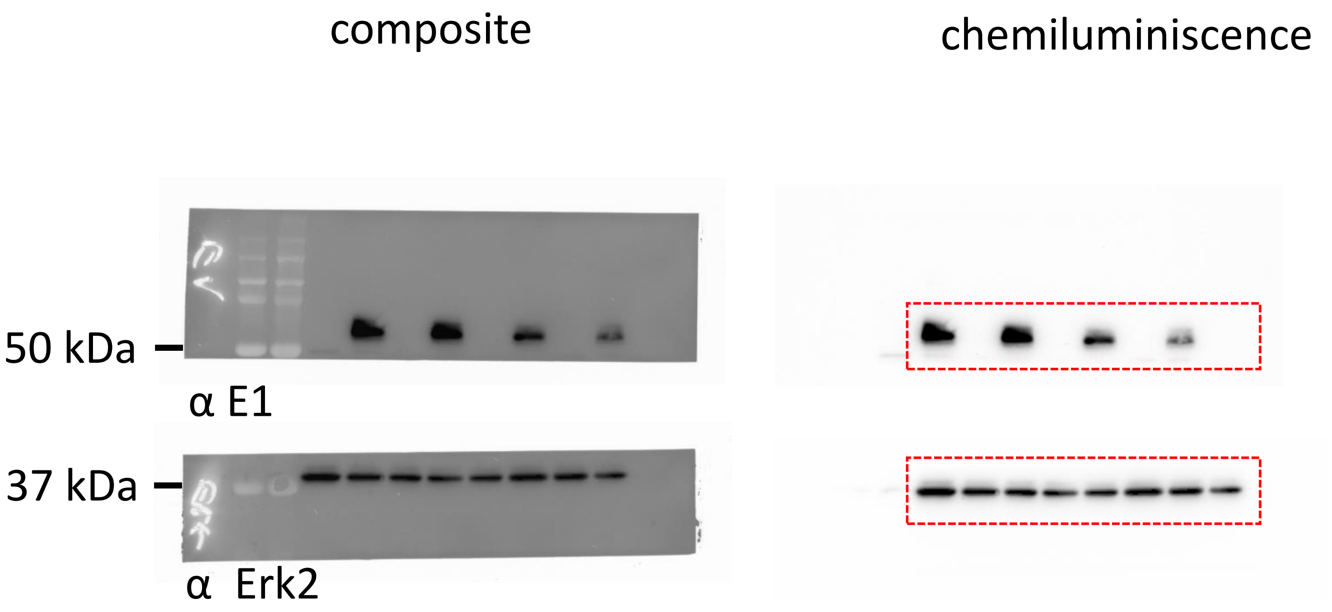

Uncropped western blot – Figure 4G

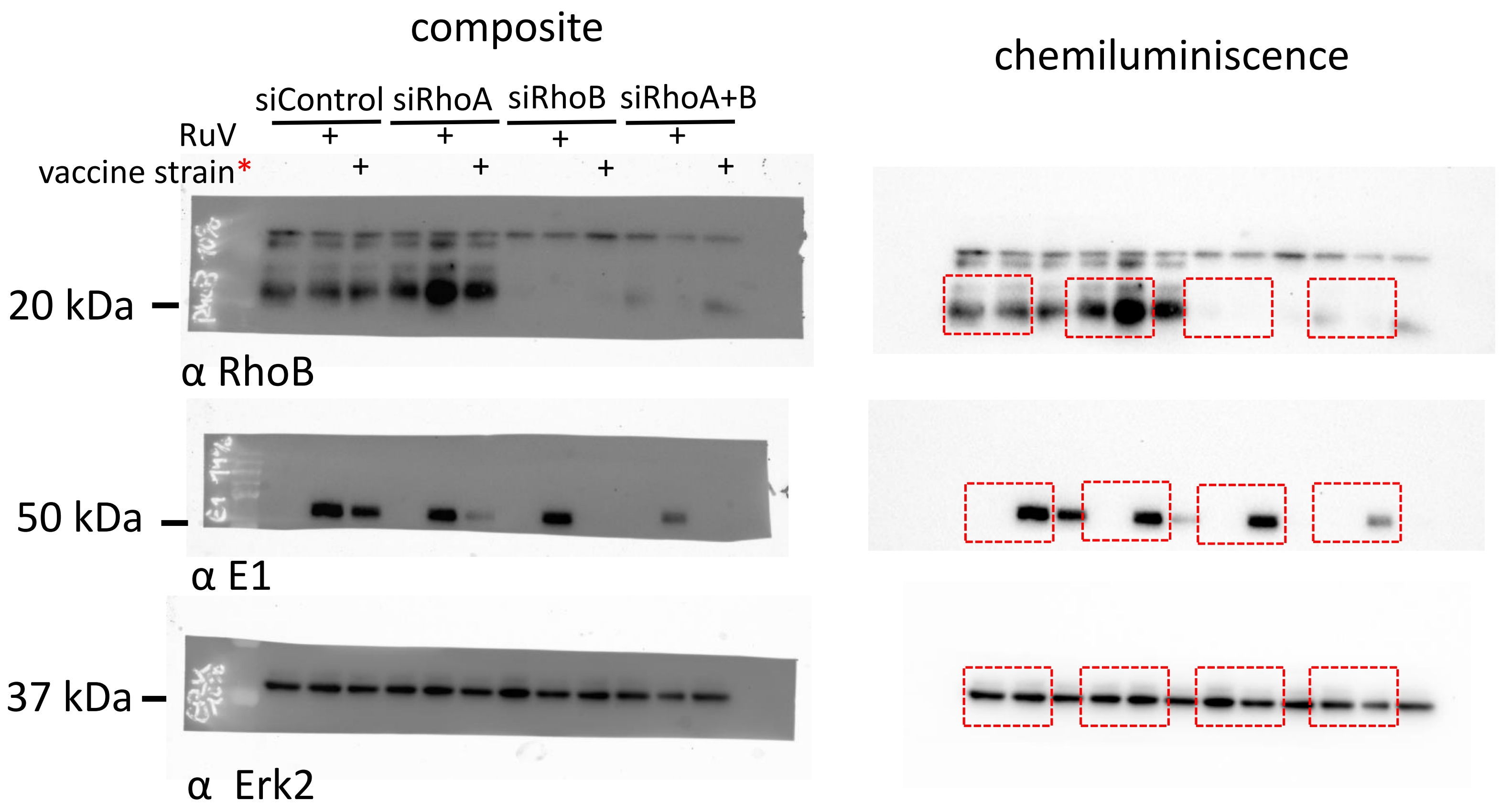

Reloaded lysates for the RhoA blot with the vaccine strain excluded.

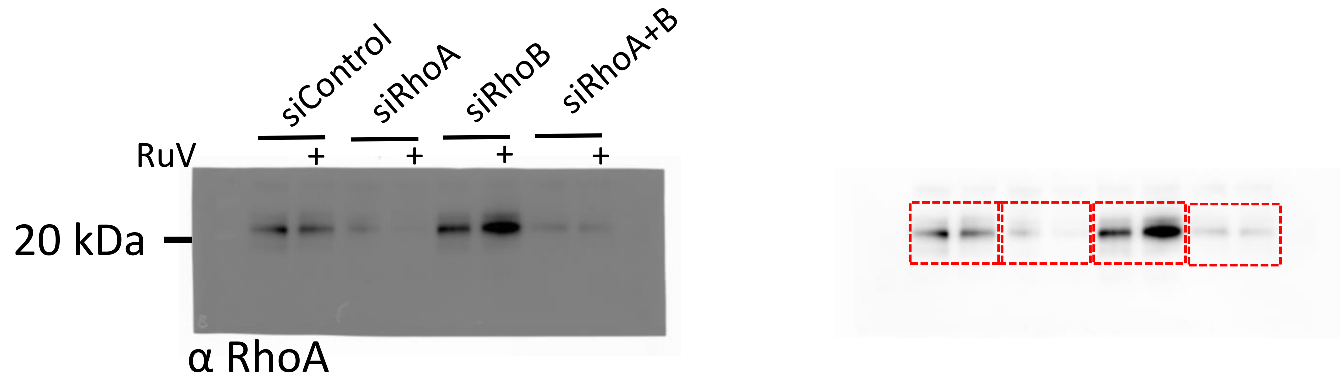

*RuV vaccine strain was not used further in the manuscript and was excluded from the subseqeunt experiments.
